# Transition Dipole Strength as a Quantitative Tool for Protein Secondary Structure Analysis

**DOI:** 10.1021/acs.jpcb.5c04203

**Published:** 2025-08-07

**Authors:** Amanda L. Cao, Lindsey M. Weissman, Lauren E. Buchanan

**Affiliations:** Department of Chemistry, 5718 Vanderbilt University, Nashville, Tennessee 37235, United States

## Abstract

Proteins adopt complex structures through dynamic folding processes that are challenging to capture experimentally. This study advances our understanding of the structural information that can be obtained by transition dipole strength (TDS) analysis, an innovative extension of two-dimensional infrared (2D IR) spectroscopy. By systematically characterizing the TDS of model α-helical peptides, we find a linear correlation between TDS values and helical length that can be used to extrapolate the maximum α-helical lengths in globular proteins, even when multiple helices are present. In contrast, the interpretation of TDS values for β-sheet structures is complicated by their increased structural diversity. While TDS is generally expected to increase with the number of β-strands, we find that actual values can vary greatly depending on higher order structural organization, including the three-dimensional folding of the peptide chain and the formation of protein complexes. This work demonstrates TDS analysis as a promising method for the elucidation of structural dynamics that cannot be obtained by other methods, especially in complex protein architectures, while highlighting the need for an increased understanding of the interplay of higher order structural organization with vibrational delocalization.

## Graphical abstract

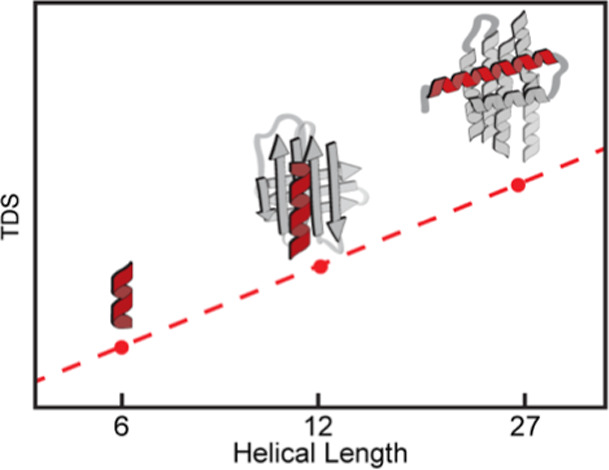

## Introduction

The intrinsic relationship between protein structure and function underpins much of molecular biology. Protein structure is hierarchically organized: secondary structures emerge from specific hydrogen bonding patterns between backbone amide groups which then further fold into complex three-dimensional, or tertiary structures, or higher order quaternary structures involving multiple protein chains. Collectively, α-helices and β-sheets account for ∼50% of residues found in folded proteins.^1^ Of these, α-helices are the most common secondary structures and are frequently found in transmembrane domains of membrane proteins^2^ or in heme-associated α-helical bundle assemblies.^3^ β-Sheets ranging from β-hairpins to β-barrels are found in a variety of native protein structures and extended β-sheets are the major structural element in amyloid fibrils.^4^

Proteins achieve their diverse functions through structural transformations, yet traditional high-resolution structural techniques often fail to capture these dynamics. Infrared (IR) spectroscopy can be employed with the requisite temporal resolution and can distinguish secondary structures by the characteristic frequency shifts produced by vibrational coupling of the backbone amide groups. For example, amide I′ frequency typically ranges from 1635 to 1660 cm^–1^ for α-helices and 1615 to 1640 cm^–1^ for β-sheets (with an additional feature around 1670–1685 cm^–1^ for antiparallel β-sheets).^5^ IR studies of protein structure have historically utilized methods such as second derivative tests,^6^ Fourier self-deconvolution curve fitting,^7^ and principal component analysis^8^ to resolve details of complex protein structures with congested spectral features.

Two-dimensional infrared (2D IR) spectroscopy has additional advantages over linear IR, including improved spectral resolution and the ability to observe crosspeaks between coupled vibrational modes, that enable more in-depth structural analysis.^9,10^ Transition dipole strength (TDS) analysis, an extension of 2D IR spectroscopy, leverages the nonlinear scaling of 2D IR signals to quantify the delocalization of the amide I′ mode across coupled residues. The transition dipole strength of a molecule ( $\vec{\mu }$ ) is related to the molecular extinction coefficient, ε, in Beer’s law (*A* = ε × *l* × *c*); linear (1D) signals scale as ${\left|\vec{\mu }\right|}^{2}$ while 2D signals scale as ${\left|\vec{\mu }\right|}^{4}$ . Thus, taking the ratio of 2D to 1D IR signals generates the TDS independent of the sample concentration or beam path length/overlap volume.^11^ Recent studies have demonstrated the sensitivity of TDS analysis in distinguishing protein structures with overlapping frequency ranges^12^ and resolving subtle conformational differences not apparent in traditional 2D IR spectra.^13–16^ However, these studies have focused on proteins that adopt primarily a single secondary structure. To expand the utility of TDS analysis to more complex protein structures with multiple structural motifs, we systematically characterize the TDS of small model peptides representing α-helix and β-sheet structures. We report a linear correlation between TDS values and helical length for model α-helices. The observed trend extends to larger globular proteins, allowing us to extrapolate the maximum helical length in lysozyme, myoglobin, β-lactoglobulin, and even other helices in the literature. In contrast, β-sheet TDS values are heavily influenced by their structural diversity. In general, TDS increases with the number of β-strands due to an increased number of coupled oscillators, in agreement with the results for α-helices in this work and for extended parallel β-sheets found in amyloid fibrils as reported in the literature.^11–13^ Notably, there is no measurable difference between the TDS of antiparallel or parallel β-strand orientations. However, comparison of two de novo model β-hairpins suggest that higher order structural factors, such as the twisting angle between strands, also influence the TDS. TDS spectra of globular proteins with significant β-sheet content exhibit a complexity that likely arises from their varied tertiary structures. These findings establish the potential of TDS as a valuable tool for quantifying elements of secondary structure within complex protein architectures, while highlighting the need for greater understanding of the interplay between secondary and tertiary organization.

## Materials and Methods

### Materials

Unless otherwise indicated, all chemicals were purchased from Fisher Scientific (Fair Lawn, NJ, USA) and used without any modifications. All standard *N*_α_-9-fluorenylmethoxycarbonyl (Fmoc)-protected amino acids, Rink Amide ProTide resin, and Oxyma were purchased from CEM Corporation (Matthews, NC, USA). *N*,*N*′-Diisopropylcarbodiimide (DIC) was purchased from Oakwood Chemicals (Estill, SC, USA). Fmoc-d-proline-OH was purchased from Chem-Impex International (Wood Dale, IL, USA) and Fmoc-Glu-OAllyl was purchased from Combi Blocks (San Diego, CA, USA). D_2_O (99%), trifluoroacetic acid (TFA), hen egg-white lysozyme (HEWL), equine heart myoglobin (Myo), and jack bean concanavalin A (ConA) were purchased from Sigma-Aldrich (St. Louis, MO, USA). β-Lactoglobulin (BLG) was purchased from A2B Chem (San Diego, CA, USA).

### Peptide Synthesis and Purification

Model α-helix and linear β-hairpin model peptides were synthesized using standard solid-phase Fmoc chemistry and DIC/Oxyma activation with a CEM Liberty Blue microwave peptide synthesizer. All peptide sequences used can be found in the Supporting Information in Table S1. Peptides are acetylated at the N-terminus and amidated at the C-terminus unless otherwise indicated. Peptides were globally deprotected and cleaved from the resin with 95% TFA, 2.5% triisopropylsilane and 2.5% DI water, washed with cold diethyl ether, and purified with reversed phase HPLC (Ultimate 3000, Thermo Fisher, Waltham, MA, USA) on an XBridge C18 preparatory column (Waters, Milford, MA, USA). Purity was checked with electrospray ionization mass spectrometry (Orbitrap XL, Thermo Fisher). Purified peptides were lyophilized to a dry powder and stored at −20 °C.

Cyclic β-sheet peptides were also synthesized using a modified version of the solid-phase peptide synthesis (SPPS) described above. An antiparallel macrocycle was synthesized by first attaching Fmoc-Glu-Oallyl to the Rink Amide ProTide resin, then adding the rest of the peptide sequence using standard SPPS methods. After addition of the last residue, the allyl group of Glu-Oallyl was removed using a Pd(0) catalyst and microwave method adapted from CEM.^17^ Finally, the N-terminal Fmoc was deprotected and on-resin cyclization was achieved using a microwave-assisted coupling method with DIC and Oxyma. A parallel macrocycle was synthesized using N- and C-terminal linkers reported by Freire and Gellman.^18^ The N-terminal linker (HO-succinic-OTMSE) and C-terminal linker (Alloc-Glu-Dpro-1,2-diamino-1,1-dimethyl) were synthesized without any modifications. The C-terminal linker was attached to the Rink Amide resin and the first strand of the macrocycle was synthesized using SPPS methods. The alloc group on the C-terminal linker was removed using Pd(0) catalyst and the same microwave method mentioned above, which allowed the second peptide chain to be synthesized. Finally, the N-terminal TMSE group was deprotected using tetrabutylammonium fluoride in tetrahydrofuran for 4 h and on-resin cyclization was achieved using the same coupling method as for the antiparallel macrocycle to form the succinyl-glycyl N-linker. Macrocycles were cleaved from the resin and purified using the same methods as linear peptides.

### Circular Dichroism Spectroscopy

All CD experiments were collected using a Chirascan VX Spectrapolarimeter (Applied Photophysics, Leatherhead, UK). Samples were collected at 21 °C in a demountable 0.01 cm path length quartz cuvette. The final concentration for each sample was 1 mM of peptide in 10 mM phosphate buffer at pH 7.6. Three spectra in the far-UV region (190–250 nm, 1 nm increments) were collected and averaged before converting to mean residue ellipticity (MRE, [θ]) according to eq 1

1 $$\left[\theta \right]\left(\frac{{\deg\times cm}^{2}}{dmol}\right)=\frac{\theta }{l\times C\times n}$$

where θ is the measured ellipticity in millidegrees, *l* is the path length in mm, *C* is the concentration in mol/L, and *n* is the number of residues.^19^ The experimental MRE at 222 nm ([θ]_222_) can be compared to theoretical maximum MRE at the same wavelength ([θ]_H_) to determine percent helicity according to eq 2

2 $$\%\;helicity=\frac{{\left[\theta \right]}_{222}}{{\left[\theta \right]}_{H}}\times 100\%$$

The theoretical maximum MRE ([θ]_H_) for a perfectly helical peptide with n residues is calculated according to eq 3

3 $$\theta_{H}=-40,000\times \left(1-\frac{x}{n}\right)+100\times T$$

where *x* is an empirical constant that corrects for non-hydrogen bonded carbonyls and *T* is temperature in °C.^20^ The constant *x* is length dependent and generally ranges from 0 to 3; here, we use a value of 2.5 as our peptides range from 8 to 23 residues in length.

### Two-Dimensional Infrared Spectroscopy

All peptide samples for 2D IR underwent hydrogen–deuterium exchange in D_2_O to deuterate the backbone amides. Peptides were then lyophilized and redissolved in 20 mM deuterated Tris buffer (pD 7.6). Model α-helical peptides were run at a final concentration of 2 mM, while β-hairpin peptides were run at 5 mM. HEWL, Myo, and BLG samples were prepared at 1 mM (or 0.13 mM for BLG) and ConA at 0.4 mM.

A detailed description of the instrumentation and data collection for 2D IR spectroscopy has been published previously.^21,22^ Briefly, a Ti:sapphire regenerative amplifier (Solstice, Spectra Physics, Milpitas, CA, USA) pumped an optical parametric amplifier (TOPAS, Light Conversion, Vilnius, Lithuania) to produce 6.1 μm mid-IR light (20 μJ, 1 kHz, ∼80 fs) through difference frequency generation. The mid-IR pulses were directed into a commercial 2D IR spectrometer (2DQuick IR, PhaseTech Spectroscopy, Madison, WI, USA) where the beam was split between the pump (90%) and probe (10%) paths. A germanium acoustic optical modulator was used to produce pairs of pump pulses with variable time delays from 0 to 2544 fs in 24 fs steps, while the probe beam was used without modification. Pump and probe pulses were focused on the sample and the resulting 2D IR signal was dispersed onto a MCT array detector. Spectra were collected without a delay between pump and probe pulses and with parallel polarization. All spectral processing and analysis were performed in MATLAB using custom scripts.

### Transition Dipole Strength Measurements with AirPLS

Methods for calculating TDS spectra directly from 2D IR spectra according to eq 4 have been described previously.^11–13,23^

4 $$d\left(\omega \right)=\frac{\frac{{\Delta OD}_{sample\left(\omega ,\omega \right)}}{{OD}_{sample\left(\omega \right)}}}{\frac{{\Delta OD}_{calibrant}\left(\omega_{\max,}\omega_{\max}\right)}{{OD}_{calibrant}\left(\omega_{\max}\right)}}\frac{I_{pump}\left(\omega_{\max}\right)}{I_{pump}\left(\omega \right)}{\left|\mu_{calibrant}\right|}^{2}$$

The linear optical density (OD) is calculated from the transmission of the 2D IR probe pulse, while the change in optical density (ΔOD) is obtained from the diagonal intensity slice of the 2D IR spectrum. We recently reported a new method to obtain a more accurate background correction for the linear OD by using an adaptive iteratively reweighted penalized least-squares regression (airPLS) algorithm,^24^ which was employed here. All TDS spectra were generated using a custom MATLAB script that employs the airPLS algorithm. *N*-Methylacetamide (NMA), a common model for an uncoupled amide I′ unit with a TDS of 0.12 *D* ^2^, was used as the calibrant molecule. A full spectrum of the pump pulse (Figure S1), *I*_pump_(ω), was collected and used to normalize the TDS spectrum relative to the pump intensity at the peak maximum for NMA, *I*_pump_(ω_max_).

## Results and Discussion

### Dependence of Transition Dipole Strength on α-Helix Length

Previous applications of TDS analysis to α-helices have used TDS as a definitive metric to distinguish them from disordered structures.^11,12^ While the two secondary structures exhibit overlapping frequency ranges, with disordered structures absorbing around 1645 cm^–1^ while α-helices absorb between 1635 and 1655 cm^–1^ depending on their local environment, vibrational coupling within the α-helix leads to larger TDS values than the 0.12 *D* ^2^ that characterizes disordered structures. Here, we aim to move beyond binary identification and quantify the dependence of TDS on helical length.

To systematically vary the helical length, we utilize model peptides based on the de novo [EK]_*N*_ helix, where *N* is the number of repeats of the core [EAAAK] sequence.^25^ Alanine (A) has a strong helical propensity due to its small, nonpolar side chain^26^ while pairs of glutamic acid (E) and lysine (K) are spaced 4 residues apart, enabling the formation of side chain salt bridges that stabilize the α-helical structure. CD spectroscopy was used to quantify the extent of helical structure for [EK]_*N*_ peptides with *N* = 1:4 (Figure 1). Disordered peptides produce a single negative peak at roughly 198 nm while helical peptides produce two peaks at 208 and 222 nm, the latter of which is used to quantify the percent helicity.^19^ [EK]_1_, which contains only 1 repeat of the core sequence, appears to be entirely disordered; thus, it serves as a control for TDS analysis. Despite having the same core sequence as the other [EK]_*N*_ peptides, the lack of structure in [EK]_1_ is likely the result of its short length (8 residues). Although proteins can contain α-helices as small as 4 residues,^27^ a short, isolated peptide chain such as [EK]_1_ is thermodynamically unfavored to form an α-helix in solution without the aid of synthetic mimics.^28–30^ The remaining [EK]_*N*_ peptides are longer and clearly helical. Using eqs 1–3, we find that percent helicity increases with the number of repeats: [EK]_2_ is 45% helical, corresponding to an average of 5.9 residues adopting an α-helical conformation, while [EK]_3_ is 74% helical (13.2 helical residues) and [EK]_4_ is 88% helical (20.2 helical residues).

**Figure 1:**
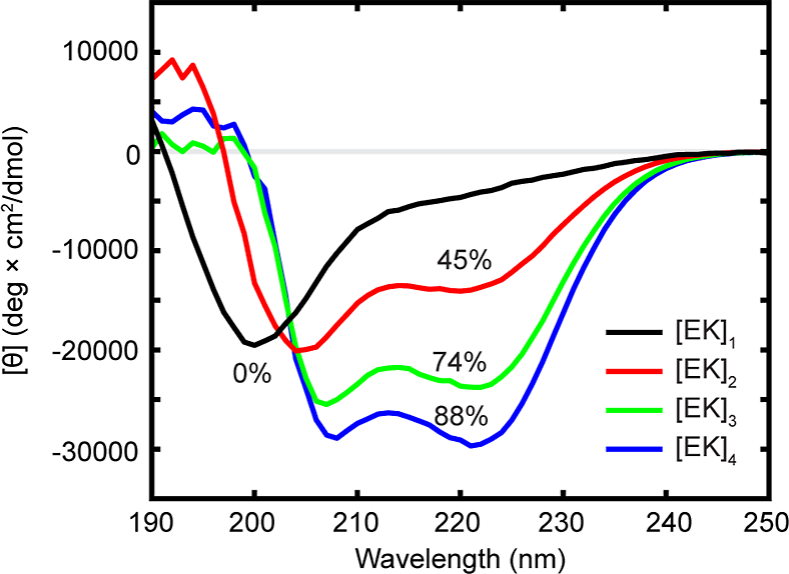
CD spectra of [EK]_*N*_ peptides indicate helicity increases with the number of repeats. Percent helicity for [EK]_2–4_ is calculated based on the 222 nm peak, while [EK]_1_ is assigned a percent helicity of 0 as the line shape indicates it is fully disordered.

2D IR spectra for the [EK]_*N*_ peptides are shown in Figure 2. [EK]_1_ exhibits a broad peak pair at 1647 cm^–1^ (Figure 2A) characteristic of a disordered peptide.^5^ An increasing redshift is observed in the amide I′ frequency of the remaining [EK]_N_ peptides (Figure 2B–D), indicative of both hydrogen bonding and increased vibrational coupling between backbone amides within an ordered peptide structure.^31–33^ These frequencies, ranging from 1637 to 1642 cm^–1^, fall within the expected range for soluble α-helical peptides.^5,33–35^ The correlation between helical length, as derived from CD, and amide I′ frequency for the [EK]_*N*_ peptides is shown in blue in Figure 2G. Frequency decreases linearly with the number of helical residues, suggesting that frequency is a reliable measure of α-helicity. However, this is only the case for short soluble peptides, such as the [EK]_*N*_ series. For globular or membrane proteins, differences in solvation and electrostatic environment can lead to a solvatochromic blueshift that opposes the redshift that arises from the organized secondary structure.^35,36^ To highlight this difference, we show 2D IR spectra for the biological proteins hen egg white lysozyme (HEWL) and myoglobin (Myo). HEWL is a 129-residue globular protein containing four α-helices, with the shortest helix being 4–8 residues in length and the longest being 12–14 residues, depending on the structural technique referenced (Figure S2A).^37–39^ Based on the length of the helices, we might expect HEWL to have an amide I′ peak around 1640 cm^–1^, similar to [EK]_2_ or [EK]_3_. However, HEWL exhibits a peak pair at 1652 cm^–1^ (Figure 2E), at a higher frequency than the disordered [EK]_1_. Myo, a 153-residue globular protein containing eight α-helices ranging from 6 to 27 residues in length (Figure S2B),^40^ might be expected to appear at a lower frequency than even [EK]_4_ but produces a peak pair at 1651 cm^–1^. Clearly, these globular proteins do not follow the linear correlation in frequency observed for the [EK]_*N*_ peptides (Figure 2G).

**Figure 2:**
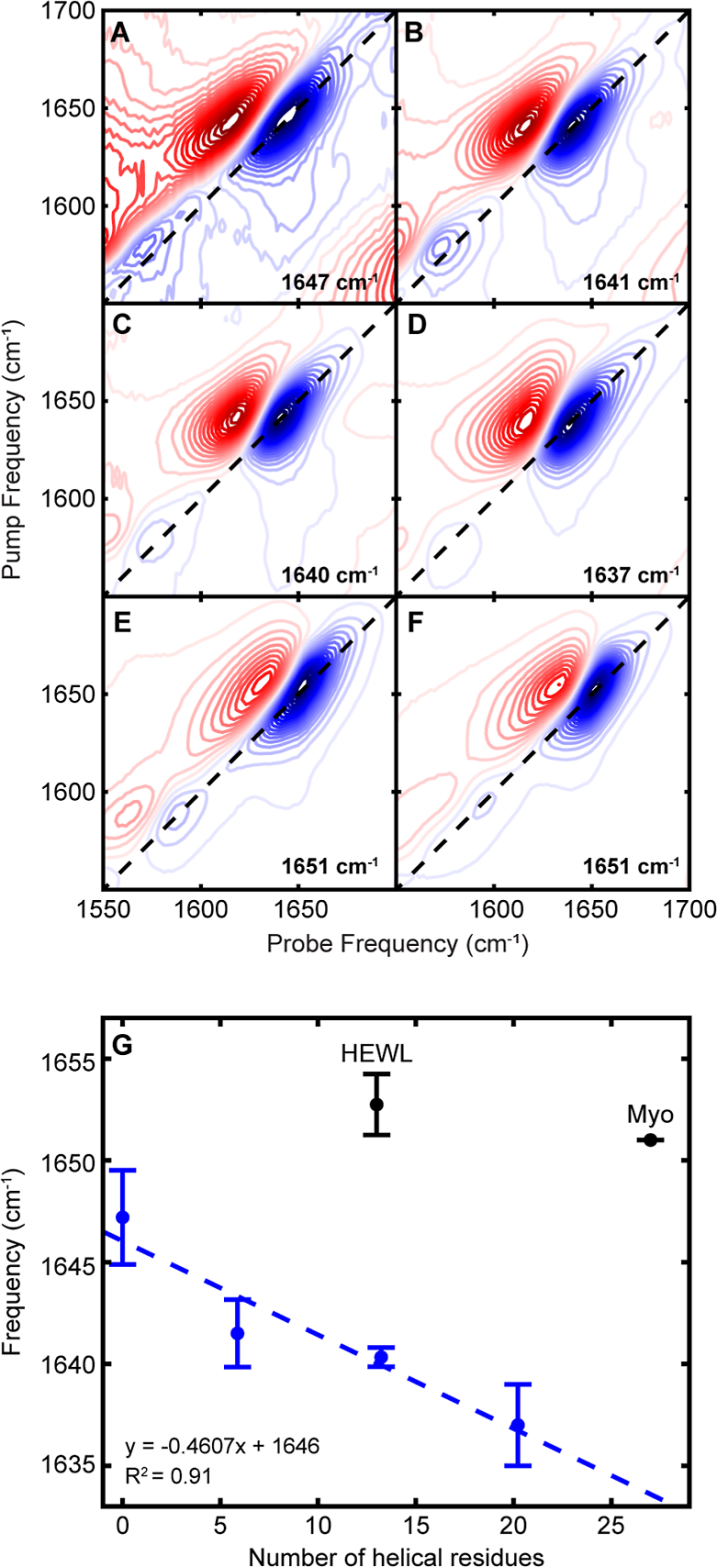
2DIR spectra for model helical peptides (A) [EK]_1_, (B) [EK]_2_, (C) [EK]_3_, and (D) [EK]_4_, as well as predominantly α-helical globular proteins (E) HEWL and (F) Myo. The frequency of the amide I′ peak (1635–1655 cm^–1^) is given on each spectrum, while peak pairs below 1600 cm^–1^ arise from sequence-dependent IR-active side chains. (G) Correlation between number of helical residues and amide I′ frequency for model [EK]_*N*_ peptides (blue) and globular proteins (black). The model peptides were fit to a trendline (dashed blue). HEWL and Myo were placed along the *x*-axis according to the number of residues in their longest α-helix. Each data point is the average frequency with errors bars representing standard deviation over *n* = 3–6.

Given that frequency alone is not a reliable indicator of α-helical structure, researchers have sought other methods of definitively identifying α-helices with IR spectroscopy. TDS analysis has previously been shown to distinguish between disordered and helical conformations of rat islet amyloid polypeptide that otherwise appeared identical in both linear and 2D IR spectra.^12^ We calculated the TDS of the four model [EK]_*N*_ peptides according to eq 4 and found a positive linear correlation with helical length (Figure 3, red). The TDS of [EK]_1_ was 0.13 ± 0.005 *D* ^2^; this is within range of the TDS for an uncoupled amide I′ mode (0.12 *D* ^2^) and thus confirms that [EK]_1_ is largely disordered. [EK]_2_ has only 5 helical residues, or barely more than a full α-helical turn. Thus, while it does exhibit a redshift in the amide I′ due to the formation of hydrogen bonds between backbone amides,^33^ the overall vibrational delocalization remains quite small, resulting in a TDS of 0.15 ± 0.01 *D* ^2^. [EK]_3_ and [EK]_4_ form longer α-helices comprising 4 and 6 turns, respectively, and their TDS are correspondingly higher at 0.21 ± 0.02 *D* ^2^ for [EK]_3_ and 0.26 ± 0.03 *D* ^2^ for [EK]_4_.

**Figure 3:**
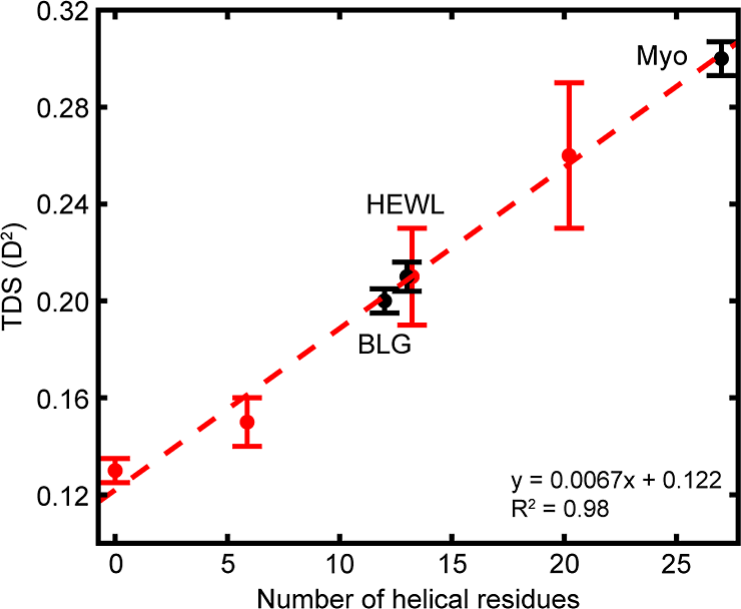
Correlation between number of helical residues and TDS for model [EK]_*N*_ (red) and globular proteins (black). The model peptides were fit to a trendline (dashed red). HEWL, Myo, and BLG were placed along the *x*-axis according to the number of residues in their longest α-helix. Each data point is the average TDS with errors bars representing standard deviation over *n* = 3–6.

While solvation strongly influences vibrational frequency,^35,36^ the TDS of a vibrational mode is unaffected by differences in local environment (Figure S3). Thus, while amide I′ frequency is an unreliable measure of α-helical content, we find that the positive correlation between TDS and helical length holds for globular proteins (Figure 3, black). A TDS of 0.21 ± 0.006 *D* ^2^ was calculated for HEWL. Based on the linear fit of the model [EK]_*N*_ peptides, this TDS predicts a helical length of 13.1 residues. While this number may initially seem low considering that HEWL contains multiple helices, vibrational delocalization can only occur over regions of continuous structure and TDS is thus limited by the lengths of the individual α-helices and not the total number of helical residues. In fact, the predicted helical length of 13.1 residues corresponds to the number of residues in the longest of HEWL’s four α-helices. Similarly, the calculated TDS for Myo, 0.30 ± 0.007 *D* ^2^, predicts a helical length of 26.6 residues which corresponds to the longest of Myo’s eight α-helices. These results indicate that for proteins where the spectral contributions from individual structural elements cannot be resolved, the apparent TDS is not additive or averaged over multiple structures. For example, if we were to predict the TDS of each of Myo’s eight α-helices based on our linear fit model, the average TDS for Myo would be 0.24 *D* ^2^, significantly lower than the measured 0.30 *D* ^2^. Instead, TDS must be determined by the greatest delocalization length and directly reports the longest α-helix present even in complex globular proteins.

### Transition Dipole Strengths of Antiparallel and Parallel β-Strands

β-Sheets are made of multiple β-strands held together by interstrand hydrogen bonds between backbone amide groups. The strands can be arranged in an antiparallel or parallel strand direction, with antiparallel being more common in globular proteins^41^ while parallel strand alignments dominate in protein aggregates such as amyloid fibrils.^42^ Here, we use model peptides with the same core sequence to evaluate the role of β-sheet size, stability, and strand alignment on TDS.

β-Hairpins, which comprise two antiparallel β-strands linked together by a loop or turn, are an attractive model system for β-sheets due to their diverse structural design and improved solubility over extended β-sheet structures. Using the same β-strand sequences identified to stabilize macrocyclic β-sheet peptides by Freire and Gellman,^18^ we designed two variations of a β-hairpin. Proline and glycine are often incorporated in de novo β-hairpin sequences to form the β-turn between strands.^43^ Our first β-hairpin design used ^L^Pro-Gly (LPG) to initiate the β-turn but the resulting LPG peptide did not form a β-hairpin, as indicated by a broad amide I′ transition in the 2D IR spectrum at 1649 cm^–1^ characteristic of structural disorder (Figure 4A).^5^ In contrast, using ^D^Pro-Gly (DPG) to initiate the β-turn produced a redshift to 1641 cm^–1^ (Figure 4B), indicating vibrational coupling in the amide backbone between adjacent strands of the folded β-hairpin. The difference in DPG and LPG peptide structure was confirmed with CD (Figure S4). We attribute this difference to the nonproteinogenic ^D^Pro side chain having a restricted φ favoring a type II’ β-turn torsion angle that strongly promotes β-hairpin formation.^44–46^ The amide I′ mode for DPG is sufficiently broad that it extends into the high frequency range where a weaker amide I′ mode would be expected for antiparallel β-sheets, which prevents that feature from being resolved although the width of the overtone (red) peak around 1682 cm^–1^ suggests the presence of a crosspeak.

**Figure 4:**
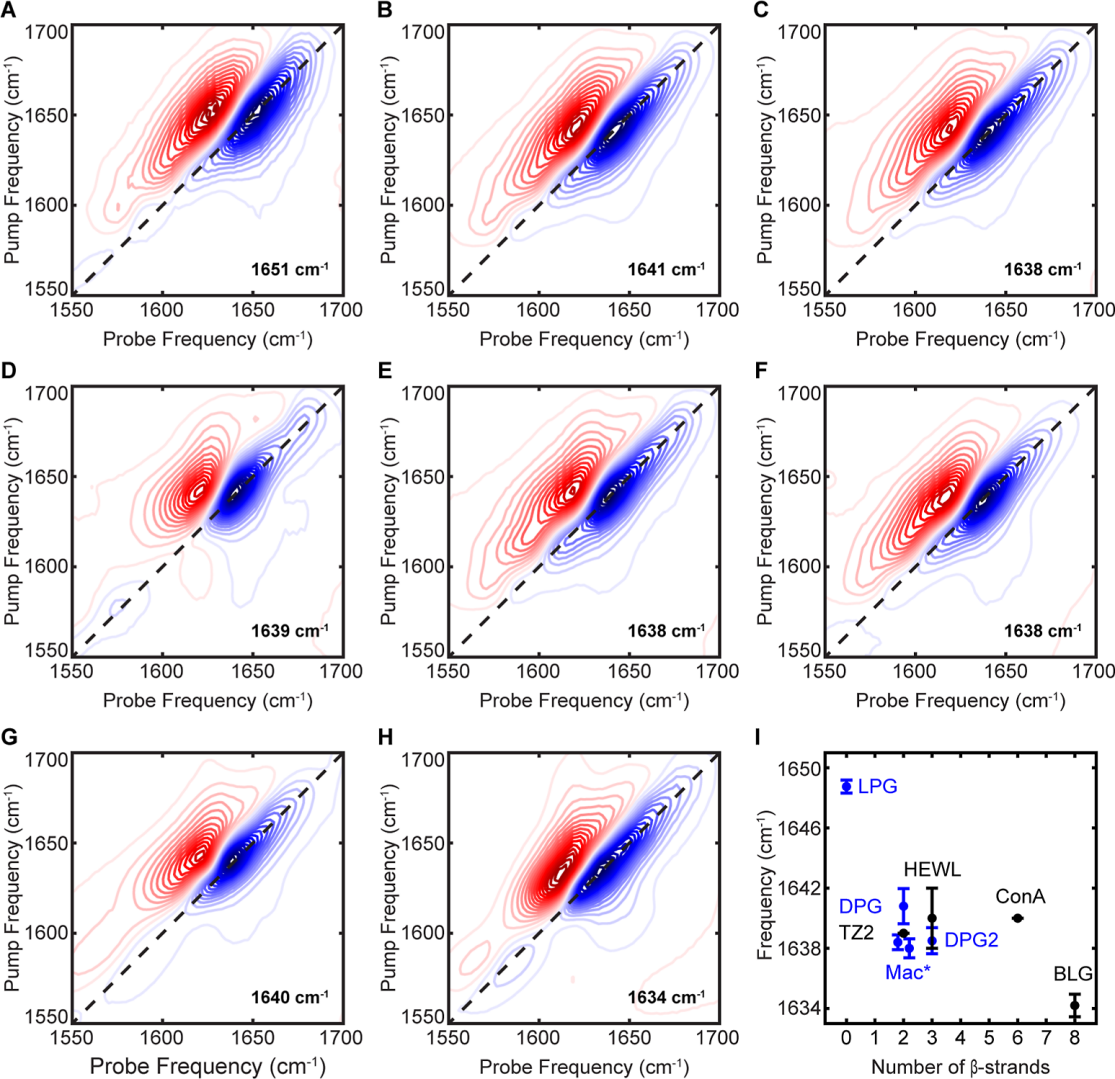
2D IR spectra of model β-sheet peptides (A) LPG, (B) DPG, (C) DPG2, (D) TZ2, (E) APmac, and (F) Pmac as well as predominantly β-sheet globular proteins (G) ConA and (H) BLG. The frequency of the main β-sheet amide I′ peak is given on each spectrum. Peptides in panel A–C, E, and F contain Pro-Gly turns that produce a weak tertiary amide I′ signature around 1612 cm^–1^, while peak pairs below 1600 cm^–1^ arise from sequence-dependent IR-active side chains. (I) Plot of frequency versus the number of β-strands does not show a linear dependence. All PG-based peptides are shown in blue and all other sequences are denoted in black. The macrocycle data points (mac*) are slightly offset for better visualization. Each data point is the average frequency with errors bars representing standard deviation over *n* = 3–5.

β-Sheet structures can extend far beyond two strands, so a third strand was added to our β-hairpin model using another ^D^Pro-Gly to create a second β-turn.^47–49^ The triple-strand peptide, DPG2, has an amide I′ frequency of 1638 cm^–1^ (Figure 4C), 3 cm^–1^ lower than DPG. This redshift indicates stronger vibrational coupling, which is typically indicative of an increased number of β-strands coupled together^50^ but other structural factors can affect the frequency of β-sheet proteins. For example, Trpzip2 (TZ2) is a de novo β-hairpin stabilized with two pairs of cross-strand tryptophan residues.^51–53^ Despite having two strands like DPG, the strongest amide I′ mode in TZ2 appears at 1639 cm^–1^ (Figure 4D), the same frequency as the triple-stranded DPG2. This peak is also narrower than for either DPG or DPG2, allowing the 1681 cm^–1^ mode characteristic of antiparallel β-sheets to be clearly resolved. While TZ2 and DPG have similar secondary structures, the interaction between paired tryptophan side chains leads to a twisted tertiary structure unique to TZ2. These results clearly demonstrate that other structural factors must be considered when interpreting the amide I′ frequency of β-sheet proteins.

Antiparallel and parallel strand alignment is another of these structural factors to consider. To systematically compare the effects of strand alignment, we employed macrocyclic peptides based on designs previously used for NMR studies.^54,55^ The strands in the antiparallel macrocycle (APmac) are linked with ^D^Pro-Gly segments at both the N- and C-terminal ends. The parallel macrocycle (Pmac) strands were connected on the N-terminus by a flexible linker while the C-terminal ends were connected by a rigid ^D^Pro-based synthetic linker reported by the Gellman group to promote a parallel β-sheet orientation that is not typically seen in small peptides.^18,56,57^ 2D IR spectra of APmac (Figure 4E) and Pmac (Figure 4F) both display an amide I′ peak pair at 1638 cm^–1^. This frequency is more comparable to the 3-stranded DPG2 than the 2-stranded DPG, likely due to backbone cyclization that eliminates terminal fraying and thus increases vibrational coupling. While the peaks are broad in both spectra, the APmac peak pair extends further into the high frequency side of the spectrum due to the additional amide I′ mode around 1683 cm^–1^ that appears for only for antiparallel β-sheets. In summary, all the de novo β-sheet models produce amide I′ peaks ∼1638–1641 cm^–1^ with no clear dependence on strand alignment or number of strands (Figure 4I).

Designed de novo β-sheet proteins are generally size limited due to challenges in maintaining solubility and avoiding aggregation for extended β-sheet structures.^58,59^ Native proteins have evolved to support a wider variety of β-sheet sizes with diverse tertiary and quaternary structures. Concanavalin A (ConA) is a β-sheet rich protein that forms homotetramers at neutral pH, with 60–70% of its residues participating in a pair of six-stranded antiparallel β-sheets in a jelly roll-like motif (Figure S2C).^60,61^ The 2D IR spectrum for ConA shows a broad amide I′ transition centered around 1640 cm^–1^ that extends toward the weaker antiparallel mode at 1677 cm^–1^ (Figure 4G). Thus, even with larger β-sheets, the amide I′ frequency for ConA clusters with the small model β-hairpins studied here. The differing frequency trends for ConA and BLG emphasizes that additional factors must affect the amide I′ frequency. Another predominantly β-sheet protein, β-lactoglobulin (BLG), adopts a cone-shaped antiparallel 8-stranded β-barrel structure (Figure S2D, light blue), but can dimerize with an additional 2-stranded β-sheet formed at the homodimer interface (Figure S2D, pink).^62,63^ Unlike ConA, the amide I′ peak for BLG is redshifted to 1634 cm^–1^, farther than any of the other β-sheet modes in this study, with the antiparallel β-sheet mode appearing at 1685 cm^–1^. While the model β-sheet peptides were fully solvated, the globular nature of ConA and BLG means that frequency could be affected by both structural and environmental differences, leading us to examine whether TDS is a more reliable measure of β-sheet structure.

We limit TDS analysis primarily to the stronger, low frequency amide I′ mode that is present for both parallel and antiparallel β-sheets; all values are summarized in Figure 5. As this mode arises from vibrational delocalization between (perpendicular to) the β-strands, we would expect the TDS to increase with the number of strands. This holds true for the Pro-Gly-based β-hairpins: the disordered LPG peptide has a TDS of 0.13 ± 0.01 *D* ^2^, the 2-stranded DPG has a TDS of 0.19 ± 0.01 *D* ^2^, and the 3-stranded DPG2 has a TDS of 0.25 ± 0.03 *D* ^2^. However, the remaining model peptides do not support a straightforward dependence of TDS on the number of β-strands. Both APmac and Pmac have identical TDS values of 0.24 ± 0.03 *D* ^2^ and 0.24 ± 0.01 *D* ^2^, respectively; as with amide I′ frequency (Figure 4I), this is more comparable to the 3-stranded DPG2 despite comprising only 2 β-strands like DPG. As structural order increases vibrational delocalization while disorder decreases it,^13^ we attribute the relatively higher TDS values for the macrocycles to their increased structural rigidity^18,55^ compared to the more flexible β-hairpins which are likely to experience terminal fraying. Notably, there is no difference in the TDS values for Pmac and APmac and negligible difference in their amide I′ frequencies. Thus, the presence of a higher frequency amide I′ mode, which arises from vibrational delocalization along (parallel to) the β-strands, with crosspeaks to the main amide I′ mode remains the only way to definitively identify antiparallel β-sheets in 2D IR spectra.^64^

**Figure 5:**
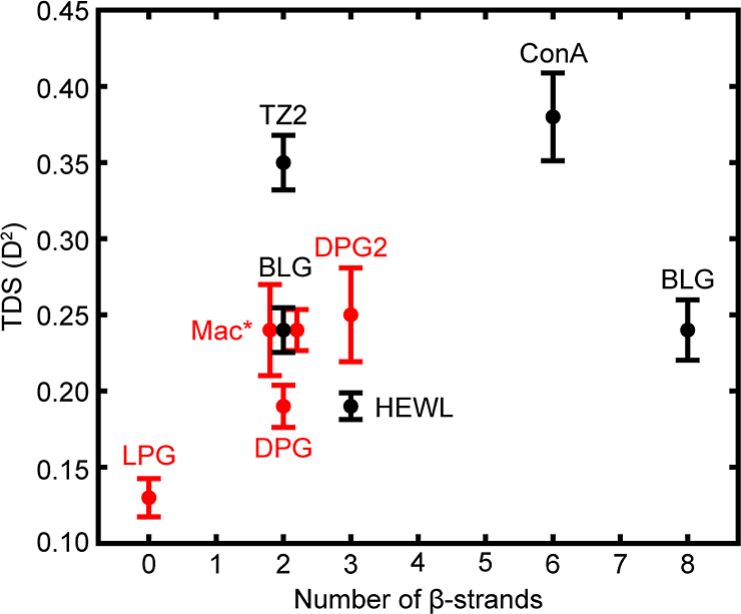
TDS of β-sheet containing peptides and proteins based on the number of β-strands. All Pro-Gly based peptides are denoted in red and other sequences are in black. There is no clear correlation between TDS and the number of β-strands. Macrocycle data points, indicated by mac*, are offset for better visualization. Each data point is the average TDS with error bars representing standard deviation over *n* = 3–5.

Of the small de novo β-sheets, TZ2 presents the largest deviation from any straightforward dependence of TDS on the number of β-strands with a TDS value of 0.35 ± 0.02 *D* ^2^. This unusually high TDS value suggests a highly ordered structure for such a small peptide, which is supported by both its relatively narrower amide I′ peak (Figure 4D) and reports of its extraordinary stability more similar to larger proteins.^51^ This stability is generally attributed to cross-strand interactions between the indole rings of two pairs of tryptophan residues at both the turn and termini ends of the β-strands,^53,65^ although it is surprising that these side chain interactions could provide greater rigidity than the backbone cyclization of Pmac and APmac. It is possible that the twisted tertiary structure of TZ2 could affect the vibrational delocalization as well as the amide I′ frequency,^50^ although the precise nature of this effect requires more investigation.

Analysis of globular proteins further highlights the complexity of TDS analysis for β-sheets. We calculated a TDS value of 0.38 ± 0.03 *D* ^2^ for 1640 cm^–1^ mode of ConA; while this is the highest TDS observed in this study, it is barely larger than the value obtained for TZ2 despite arising from multiple 6-stranded β-sheets. Interestingly, the TDS spectrum for ConA reveals a second peak at 1625 cm^–1^ (Figure S5C), which we attribute to a distinct β-sheet mode that is not resolved in the 2D IR spectrum (Figure 4G) due to the broad line shape of the main amide I′ mode. Previous studies of ConA have suggested this low-frequency mode arises from β-sheets with stronger hydrogen bonding^66^ or ConA aggregates.^67^ If the 1625 cm^–1^ peak was caused by from β-sheet aggregates, we would expect TDS values of 0.3–1.25 *D* ^2^ as observed for other extended β-sheet aggregates such as amyloid fibrils.^13–16^ Instead, the TDS at 1625 cm^–1^ is around 0.23 ± 0.03 *D* ^2^, much lower than the main peak at 1640 cm^–1^, which supports the hydrogen-bonding hypothesis.

The TDS spectra of HEWL also reveals a low frequency mode at 1638 cm^–1^ (Figure S5A) that is obscured in the 2D IR spectrum by the predominant α-helical amide I′ transition (Figure 2E). We attribute this peak to a short 3-stranded β-sheet (Figure S2A, cyan). The TDS of HEWL’s β-sheet mode is 0.19 ± 0.01 *D* ^2^, most comparable to the 2-strand β-hairpin model (Figure 5). In the case of both ConA and HEWL, the additional vibrational modes are obscured in linear or 2D IR spectra without the use of spectral deconvolution techniques. TDS improves the sensitivity of IR spectral analysis by measuring vibrational delocalization, an intrinsic property of the protein structure.

BLG is a predominantly β-sheet globular protein with one 12-residue α-helix (Figure S2D, orange). While the α-helix appears as a poorly resolved shoulder around 1650 cm^–1^ in the 2D IR spectrum (Figure 4H), the TDS spectrum (Figure S5D) shows a clear peak with a TDS of 0.20 ± 0.005 *D* ^2^. This corresponds to a helical length of 11.6 residues according to the linear fit equation derived in Figure 3, in excellent agreement with the PDB structure. In addition to the α-helix signature, two additional peaks are observed in the TDS at 1 mM (Figure S5D). The main peak at 1634 cm^–1^ arises from the 8-stranded β-barrel. The second peak at 1626 cm^–1^ arises from BLG homodimers formed at concentrations above 0.27 mM and is attributed to an interfacial β-sheet with a single strand contributed by each of the constituent monomers.^68^ The same TDS value of 0.24 ± 0.02 *D* ^2^ was obtained for both peaks (Figure 5) despite the peaks arising from β-sheets of different sizes (8 strands versus 2 strands). Thus, while the amide I′ frequency of BLG is the most redshifted of any of the β-sheets studied here, which would typically be attributed to increased vibrational delocalization due to its larger β-sheets, TDS does not support this analysis. Instead, the TDS for both BLG β-sheet modes are comparable to the model β-hairpins. At 0.13 mM, monomeric BLG is favored although the 2D IR spectrum remains nearly identical (Figure S6A). The TDS spectrum reveals that while the structure of the 8-stranded β-barrel remains the same at lower concentrations, as indicated by the consistent TDS value of 0.24 ± 0.02 *D* ^2^, the 1626 cm^–1^ mode now appears as a weak, poorly resolved shoulder (Figure S6B). The persistence of a lower TDS peak at 1626 cm^–1^ suggests that homodimers are still present in equilibrium with monomers, but that the interfacial β-sheet is less ordered. Thus, TDS provides a more sensitive measure of the structural ordering of transient species and quaternary protein structure.

## Conclusion

This study systematically explores how TDS analysis complements IR studies by offering deeper insights into protein secondary structure. Further, our studies of globular proteins with complex secondary structures highlight the unique power of TDS spectra to resolve overlapping spectral features in both linear and 2D IR spectra. We establish a strong linear correlation between the TDS of the amide I′ mode and α-helical length for both model peptides and globular proteins such as HEWL and Myo. This contrasts with attempts to analyze the frequency of the amide I′ mode, which is an unreliable measure of α-helical structure due to spectral overlap with disordered structures and significant solvatochromism. Critically, TDS values of α-helical modes correspond to the length of the longest α-helix in the protein, even when multiple helices of varied lengths are present and not resolved in the spectra. This trend holds true for TDS values reported by other researchers in the literature, even without the increased accuracy we recently demonstrated using automated baseline correction during the TDS calculation.^24^ Grechko and Zanni reported a TDS value of 0.26 *D* ^2^ for AKA, a soluble α-helix.^11^ Based on our model, this TDS would correspond a helical length of 21 residues, in excellent agreement with its fractional helicity of 22 residues calculated from CD.^69^ In a separate study, they calculated a TDS of 0.2 *D* ^2^ for rat islet amyloid polypeptide (rIAPP) in a model membrane,^12^ which we would predict to correspond to a helical length of 12. NMR studies of membrane-bound rIAPP have identified α-helical structures spanning from residues A5–S23 with flexibility at residues R18 and S19 that distorts the helix structure and thus truncates the length of the continuous α-helix to be 13 residues, which again agrees with our model.^70,71^ The ability of TDS spectra to measure maximum α-helical length is unique from other optical spectroscopies and complementary to CD spectroscopy, which informs on the overall helicity of a protein but not the size or number of helices. In combination, CD and TDS analysis can provide a more complete picture of α-helical structures.

We sought to establish a similar relationship for the TDS of β-sheet structures. Previous TDS studies of β-sheets have focused primarily on amyloid fibrils, which are characterized by far more extensive and ordered parallel β-sheets than typical for native protein structures. This leaves a gap in our understanding of which factors influence the TDS of β-sheets. For example, it is generally assumed that vibrational delocalization scales with the number of β-strands for soluble β-sheets,^72^ but this is not the case in amyloid fibrils where the delocalization length will always be significantly shorter than the hundreds or thousands of β-strands that extend along the fibril length.^4,73^ In fact, calculations of inverse participation ratios from the TDS typically predict delocalization lengths of only 3–20 strands for amyloid fibrils and other amyloid-like aggregates;^13,23^ as a result, differences in TDS are attributed primarily to differences in structural ordering for these systems. In this study, we did not find a clear correlation between number of β-strands and TDS even for β-sheets comprising fewer than 10 strands. While a linear scaling of TDS is observed for a series of β-hairpins based on the same core sequence, this trend does not hold for other model β-sheets of similar size. For example, the TDS of the highly twisted TZ2 is nearly double that of DPG, despite both peptides forming a 2-stranded β-hairpin, and is more comparable to the 6-stranded β-sheets of the globular protein ConA. A similar phenomenon is seen in CD spectroscopy, where a twisted β-stand geometry increases the amplitude of the characteristic β-sheet bands.^74^ Further, we found no difference in the TDS of parallel versus antiparallel macrocyclic peptides, suggesting that strand orientation does not affect the delocalization of the amide I′ mode perpendicular to the strands. These results suggest that tertiary structure may play a larger role in the vibrational delocalization of β-sheets than for α-helices.

Ultimately, combined experimental and computational studies may be required to understand how the structural diversity of β-sheets results in such varied TDS. It is more challenging to calculate IR intensities than vibrational frequencies due to vibrational non-Condon effects. However, Qian and co-workers recently demonstrated a mixed quantum/classical approach that significantly improved the IR peak intensities in calculated spectra of nucleic acids which, like proteins, are highly sensitive to solvation, hydrogen bonding, and structural differences.^75^ Their methods helped to close the gap between experimental and theoretical work in understanding TDS and could logically be extended to proteins. Our findings will be critical to guiding such computational efforts and pave the way for future research into increasingly diverse structural motifs.

## Supplementary Material



## Notes

A table of peptide sequences, referenced PDB structures, and additional spectral data (PDF)
