# Supplementary material for: Transition Dipole Strength as a Quantitative Tool for Protein Secondary Structure Analysis
Source: J Phys Chem B. 2025 Aug 7;129(33):8382–91. doi: 10.1021/acs.jpcb.5c04203 (PMC12376092; doi:10.1021/acs.jpcb.5c04203)
Supplement: Supplementary file 1 [file jp5c04203_si_001.pdf]

Supporting Information

Transition Dipole Strength as a Quantitative Tool for Protein Secondary Structure Analysis

*Amanda L. Cao, Lindsey M. Weissman, Lauren E. Buchanan**

Department of Chemistry, Vanderbilt University, Nashville, Tennessee 37235, United States

*Corresponding author: lauren.e.buchanan@vanderbilt.edu

Supporting Information

- Supplementary methods
- Table S1: Sequences of model peptide
- Figure S1: Pump spectrum
- Figure S2: PDB structures of globular proteins
- Figures S3–S6: Additional spectra

SUPPLEMENTARY METHODS

Fourier transform infrared spectroscopy (FTIR)

Absorption spectra of 130 mM N-methylacetamide (NMA) in D_2O or $CHCl_3$ were collected using a Thermo Scientific Nicolet iS20 FTIR (Waltham, MA, USA) with a MCT detector. Reported spectra comprise 256 scans obtained with 16 cm^{-1} resolution. Samples were placed between CaF_2 windows with a 50 μm Teflon spacer. The amide I' peak for NMA was integrated between points where the first derivative crossed zero on either side of the peak in order to calculate the transition dipole strength (TDS) according to Eq. S1, where $|\mu|^2$ is the TDS and ε is the extinction coefficient.[1,2]

$$|\mu|^2 = 9.18\text{x}10^{-3} \int \frac{\varepsilon(\omega)}{\omega}\, d\omega \quad \text{(S1)}$$

Table S1. Sequences of model α-helix peptides and β-hairpin peptides. Lowercase p denotes D-proline.

Name	Sequence
$[EK]_N$, N=1:4	Ac-WA[EAAAK]$_N$A-NH_2, N=1:4
Lpro-Gly (LPG)	Ac-VTKFRKPGQTFYRV-NH_2
Dpro-Gly (DPG)	Ac-VTKFRKpGQTFYRV-NH_2
Dpro-Gly x2 (DPG2)	Ac-VTKFRKpGQTFYRVpGKKFVQ-NH2
Parallel macrocycle (Pmac)	cyclo(Succ-VTKFRKp-dadme-QTFYRVG)
Antiparallel macrocycle (APmac)	cyclo(GVTKFRKpGQTFYRVp)
Trpzip 2 (TZ2)	SWTWENGKWTWK-NH_2

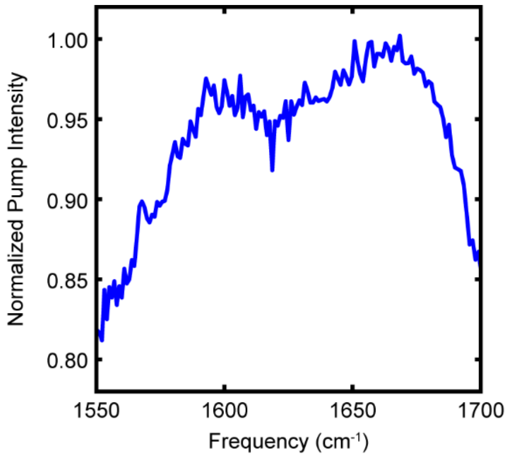

Figure S1. Spectrum of normalized pump intensity.

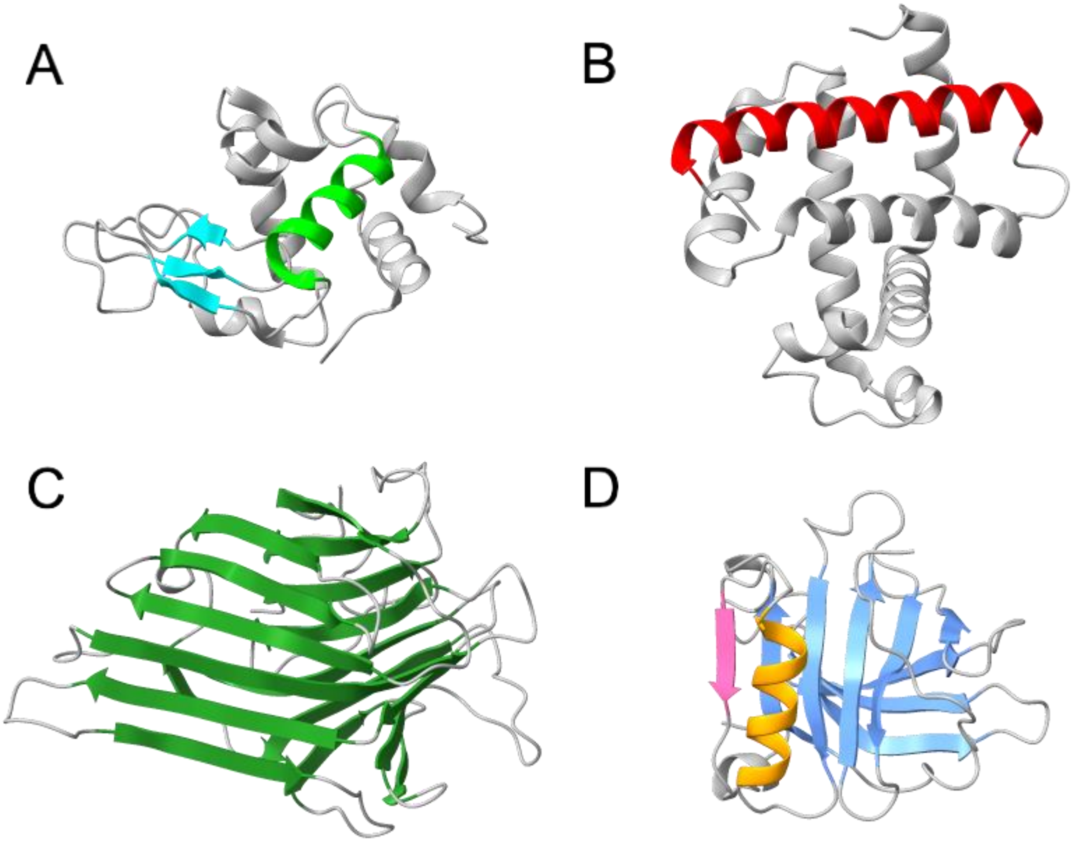

Figure S2. PDB crystal structures of representative globular proteins. A) Hen egg-white lysozyme (HEWL, PDB: 1DPX) has 4 α-helices, with the longest continuous helix (green) stretching between residues 24–36 (or 37, depending on the structural technique referenced).[3–5] Lysozyme also contains a small 3-strand β-sheet (cyan). B) Myoglobin (Myo, PDB: 1WLA) crystal structure has 6 α-helices, with the longest continuous helix (red) falling between residues 124–150.[6] C) Concanavalin A (ConA, PDB: 2CNA) has two 6-stranded β-sheets (dark green).[7] D) β-lactoglobulin (BLG, PDB: 3BLG) has an α-helix (orange) stretching between residues 130–141, 8-stranded β-barrel (light blue), and a β-strand that participates in the homodimer interface (pink).[8]

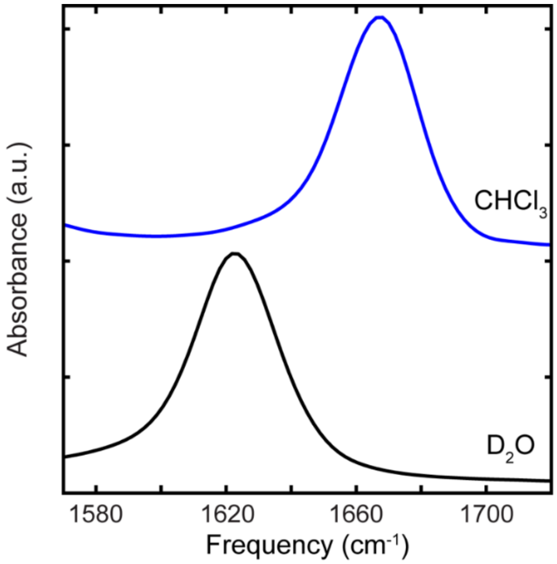

Figure S3. FTIR spectra of NMA in D_2O or $CHCl_3$. NMA in D_2O (black) has an amide I' frequency of 1623 cm^{-1} with a calculated TDS of 0.12 D^2 using Eq. S1. The frequency for NMA in $CHCl_3$ (blue) is blue shifted from D_2O to 1667 cm^{-1} but has the same calculated TDS of 0.12 D^2. Thus, while solvatochromic shifts are observed for the amide I peak of NMA, the TDS is independent of the dielectric constant of D_2O (78.1) and $CHCl_3$ (4.81).

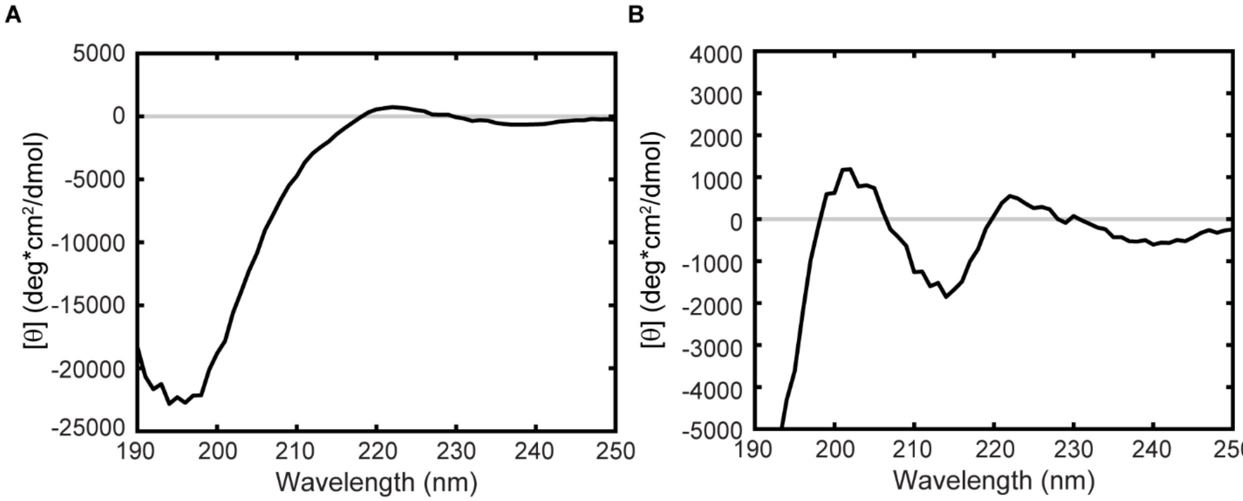

Figure S4. CD spectra for A) LPG and B) DPG. The secondary structure of LPG and DPG are different as a result of changing the stereochemistry of proline in the turn segment. LPG has a disordered structure indicated by the characteristic minimum at 198 nm. The β-sheet structure for DPG is confirmed by the negative peak at 215 nm.

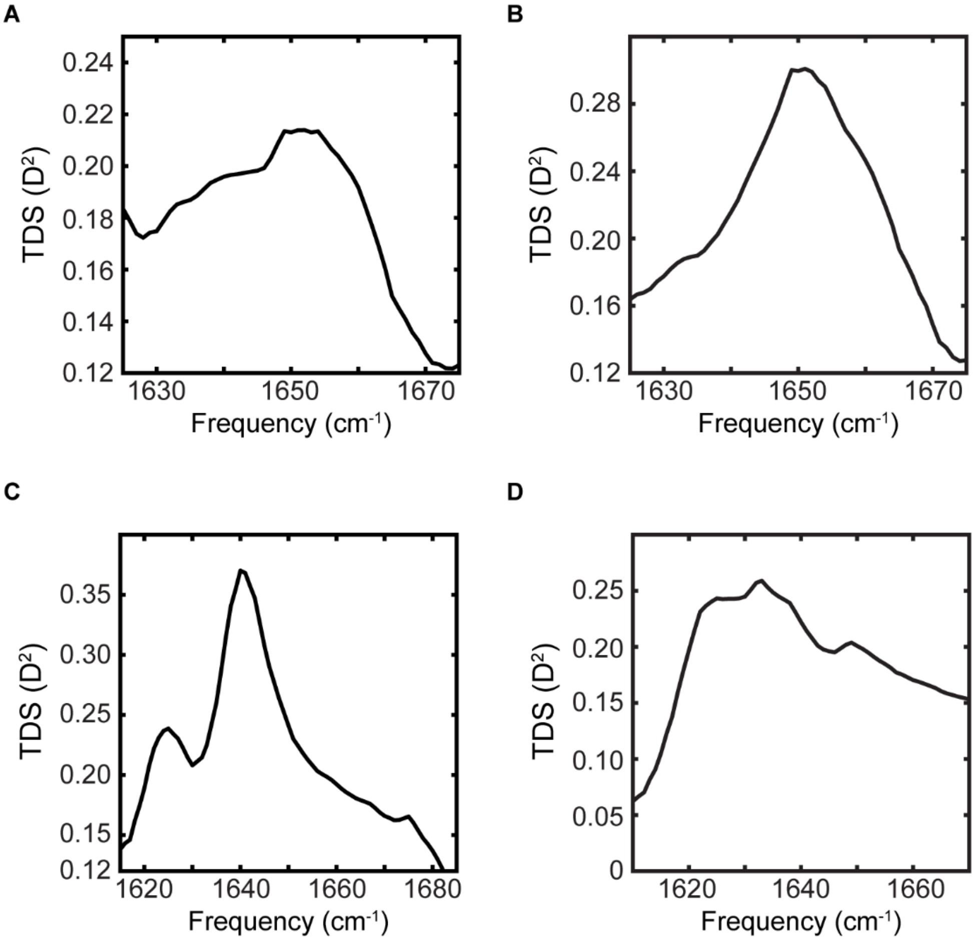

Figure S5. TDS spectra for globular proteins. A) HEWL has a broad TDS spectrum with the main α-helical peak at 1652 cm^{-1} (TDS 0.21 D^2) and minor β-sheet peaks around 1638 cm^{-1} (TDS 0.19 D^2). B) Myo exhibits a single TDS peak centered at 1651 cm^{-1} (TDS 0.28 D^2). C) The TDS spectrum of ConA shows two β-sheet peaks. The higher intensity peak at 1640 cm^{-1} (TDS 0.38 D^2) is assigned to the 6-stranded β-sheets observed in the PDB structure, while the weaker peak at 1625 cm^{-1} (TDS 0.23 D^2) is attributed to a different β-sheet structure with stronger hydrogen bonding. D) The TDS spectrum of BLG reveals an α-helical mode at 1651 cm^{-1} (TDS 0.20 D^2) and 2 β-sheet modes at 1626 cm^{-1} and 1634 cm^{-1} with, on average, identical TDS of 0.24 D^2.

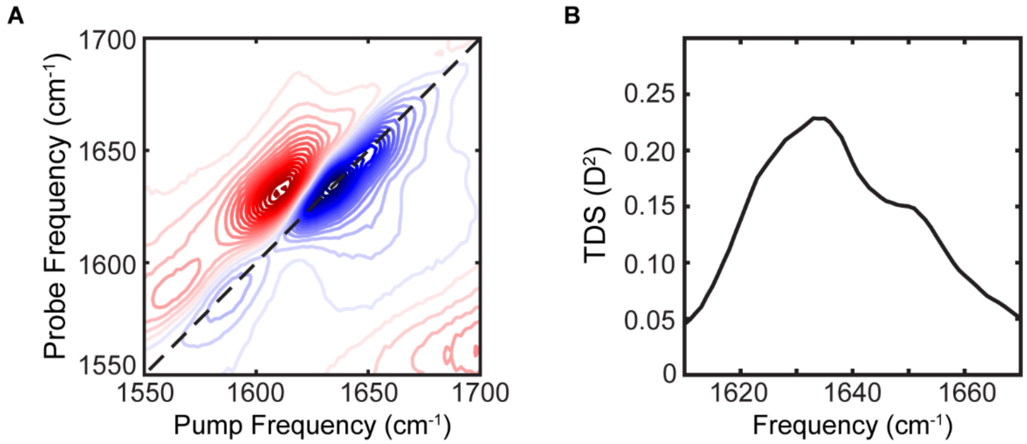

Figure S6. Spectral difference for low concentrations of BLG. A) The 2D IR spectrum of 0.13 mM BLG is similar to 1 mM BLG (Fig. 4H), although equilibrium should favor monomers at the lower concentration and homodimers at the higher concentration. B) The TDS spectrum for 0.13 mM BLG shows a much weaker β-sheet mode at 1626 cm^{-1} than observed for 1 mM BLG (Fig. S5D), suggesting that while dimers are still present, they are less ordered than at the higher concentration. There is no significant difference in the TDS for the 1634 cm^{-1} β-sheet and the 1650 cm^{-1} α-helix peak between concentrations.

REFERENCES

(1) Grechko, M.; Zanni, M. T. Quantification of Transition Dipole Strengths Using 1D and 2D Spectroscopy for the Identification of Molecular Structures via Exciton Delocalization: Application to α-Helices. *J. Chem. Phys.* **2012**, *137* (18), 184202.

(2) Ackels, L.; Stawski, P.; Amunson, K. E.; Kubelka, J. On the Temperature Dependence of Amide I Intensities of Peptides in Solution. *Vib. Spectrosc.* **2009**, *50* (1), 2–9.

(3) Smith, L. J.; Sutcliffe, M. J.; Redfield, C.; Dobson, C. M. Structure of Hen Lysozyme in Solution. *J. Mol. Biol.* **1993**, *229* (4), 930–944.

(4) Schwalbe, H.; Grimshaw, S. B.; Spencer, A.; Buck, M.; Boyd, J.; Dobson, C. M.; Redfield, C.; Smith, L. J. A Refined Solution Structure of Hen Lysozyme Determined Using Residual Dipolar Coupling Data. *Protein Sci.* **2001**, *10* (4), 677–688.

(5) Weiss, M. S.; Palm, G. J.; Hilgenfeld, R. Crystallization, Structure Solution and Refinement of Hen Egg-White Lysozyme at PH 8.0 in the Presence of MPD. *Acta Crystallogr. Sect. D* **2000**, *56* (8), 952–958.

(6) Maurus, R.; Overall, C. M.; Bogumil, R.; Luo, Y.; Mauk, A. G.; Smith, M.; Brayer, G. D. A Myoglobin Variant with a Polar Substitution in a Conserved Hydrophobic Cluster in the Heme Binding Pocket. *Biochim. Biophys. Acta - Protein Struct. Mol. Enzymol.* **1997**, *1341* (1), 1–13.

(7) Reeke, G. N.; Becker, J. W.; Edelman, G. M. The Covalent and Three-Dimensional Structure of Concanavalin A. IV. Atomic Coordinates, Hydrogen Bonding, and Quaternary Structure. *J. Biol. Chem.* **1975**, *250* (4), 1525–1547.

(8) Qin, B. Y.; Bewley, M. C.; Creamer, L. K.; Baker, H. M.; Baker, E. N.; Jameson, G. B. Structural Basis of the Tanford Transition of Bovine β-Lactoglobulin,. *Biochemistry* **1998**, *37* (40), 14014–14023.
